# COVID-19 safe dental practices among a group of Egyptian students: a cross-sectional study

**DOI:** 10.1186/s13756-025-01591-w

**Published:** 2025-07-01

**Authors:** Dena Ali Abozaid, Dina Hamed, Karim Mostafa Hindy, Mayar Moustafa Budair, Ziad Baher Hussein, Ziyad Islam Hegazy, Dina Nabih Kamel Boulos

**Affiliations:** 1 Department Community, Environmental, and Occupational Medicine, Faculty of Medicine, Ain Shams University, Cairo, Egypt; 2 School of Medicine, Newgiza University, Giza, Egypt; 3 Department of Public Health and Community Medicine, School of Medicine, Newgiza University, Giza, Egypt

**Keywords:** COVID-19, Dentists, Infection control, Safe dental practice

## Abstract

**Background:**

The COVID-19 pandemic has had a tremendous effect on dental practice. Dental practice during the pandemic has been associated with an increased risk of COVID-19 transmission. Adopting proper infection control measures in dentistry helps alleviate the risk of infection. The pandemic has created an even more challenging environment for dentists to perform their job safely, thus necessitating the adoption of very strict infection control measures for safe dental practice.

**Objectives:**

This study aimed to assess the knowledge, attitude, and practice (KAP) regarding COVID-19 safe dental measures among dentistry students currently in clinical training.

**Methods:**

A cross-sectional study was conducted. The study included a total of 104 dental students, who took clinical practical sessions in a nongovernmental dentistry school, in Egypt. Data collection was performed via an online questionnaire.

**Results:**

This study found that a total of 88.5% of the dental students had a satisfactory safe dental measures practice score. A total of 93.2% of the students had a positive attitude towards safe dental measures. Practice of hand hygiene was the most common safe dental practice among the students, and they practiced it all the time (82.7%), whereas checking patient temperature was the least common practice (28.8%). There was a statistically significant association between sex and hand hygiene (*p* = 0.023), checking patient temperature (*p* = 0.046). There was a positive moderate correlation between students’ safe dental practice score and their attitude score (*r* = 0.470, *p* < 0.001). There was a weak inverse correlation between safe dental practice score and age (*p* = 0.029, *r* = (- 0.214)).

**Conclusions, and Recommendations:**

This study highlights key strengths and gaps in dental education, reinforcing the importance of safety protocols in enhancing professional standards and safeguarding public health. The findings support the integration of pandemic-era measures into standard infection control practices. Nonetheless, specific domains—particularly temperature screening and knowledge regarding the application of HEPA filtration systems—require further emphasis and curricular enhancement.

## Background

Coronavirus disease 2019 (COVID-19) is a newly discovered viral infection that started in Wuhan, China, in December of 2019. Globally, since the start of the COVID-19 pandemic and until August 2nd, 2021, there have been more than 198 million confirmed cases of COVID-19, as well as more than 4 million deaths worldwide [1]*.* COVID-19 causes severe acute respiratory syndrome coronavirus 2 (SARS-CoV-2), a type of lower respiratory tract infection that can be severe and possibly fatal. Preventive measures to control the spread of COVID-19 include careful infection control, patient isolation, and social distancing [2].

The COVID-19 pandemic has been associated with significant occupational risk for healthcare professionals. Owing to their direct contact with patients, inhalation of aerosols released during dental treatments, and contact with contaminated surfaces, dentists are the front-line healthcare workers most at risk of contracting COVID-19 [3]*.* In Poland, not only was there a sudden drop in the number of dental procedures performed, but also dental care professionals reported experiencing confusion, fear, and anxiety [4]. There is a high risk of infection during dental practice, so it is imperative to adopt strict infection control measures [5]. Airborne contamination can be minimized by adopting proper infection control during all dental procedures [6], which highlights its importance during the COVID-19 pandemic. Previous studies conducted to evaluate infection control measures among dentists during COVID-19 pandemic in USA, Poland and Germany reported a generally strict infection control measure [7–9]. A multicentric study conducted among dental practitioners in low to middle income countries (India, Malaysia, Saudi Arabia, Thailand, Cambodia), as well as Italy reported that nearly half of the dentists felt that it was acceptable to work during the COVID-19 pandemic, as they perceived it as a low-risk situation. Additionally, most of the participants were adhered to infection control measures [10]. On the other hand, a study conducted among Jordanian dentists revealed that, although participants were aware of COVID-19 symptoms and modes of transmission, they had a limited understanding of the extra precautionary methods that would protect them and their patients from the virus, and that only 18% of them considered COVID-19 to be very dangerous [11]. In Egypt, there is scarce information and research that addresses this issue.

Previous studies on infection control practices among dental professionals and students highlight key differences from the current research. A quasi-experimental study conducted before the pandemic at a primary health care (PHC) dental clinic revealed inconsistent adherence to protective measures, with only 20% of participants consistently wearing face masks and 60% using them occasionally. However, the study was limited by its small sample size—20 dentists and 5 nurses—and its confinement to a single district PHC, which may restrict the generalizability of its findings. Additionally, the two-month intervention period raises concerns about its ability to assess long-term adherence to infection control measures [12]. Similarly, a study at Tanta University examined infection control practices among dental students, finding that adherence improved as students advanced in their academic years. More than half demonstrated satisfactory infection control practices, yet the study did not specify whether new dental safety curricula were introduced during the pandemic. Moreover, while acknowledging the university’s hybrid teaching model, it lacked detailed information on the structure of clinical training sessions, including whether students engaged in hands-on practice [13]. These limitations underscore the need for further investigation into the impact of education and training methodologies on infection prevention in dental settings. The current study aimed to assess, students’ knowledge of COVID-19 safe dental measures, attitude towards infection control measures and their perception of the effectiveness of these measures, as well as to measure the frequency of practicing"COVID-19 safe dental measures"among dentistry students. The students in the current study were actively engaged in clinical training, regularly attending dental sessions at the NGU School of Dentistry. Under the supervision of faculty and instructors, they practiced and managed patients, gaining hands-on experience in a controlled academic environment. This study highlights key strengths and gaps in dental education, reinforcing the importance of safety protocols in enhancing professional standards and safeguarding public health. Its findings can contribute to refining the existing curriculum, helping to mitigate risks in dental settings while promoting the long-term well-being of both patients and practitioners.

## Subjects and methods

### Study design and study settings

A cross-sectional study was conducted at the school of dentistry at Newgiza University (NGU), NGU is a newly established multidisciplinary nongovernmental private university in Egypt, with a partnership with University College London (UCL). NGU education is marked by a strong focus on academic excellence and practical knowledge and ability to deliver a high-quality and contemporary undergraduate dental program, making the NGU dental school a unique experience of learning and an appropriate place to assess infection control and safe dental practices during the COVID-19 pandemic, as the university is very well equipped. The data collection started in 2021 and was completed by the end of 2022.

### Study population

This study was conducted among NGU dentistry school students. Students in their clinical training and take dental clinical sessions in the NGU school of dentistry regularly, where they practice and manage patients under the supervision of school staff and instructors, were included in the study. First and second-year students were excluded from the study as they did not have clinical sessions. As part of their regular dentistry curricula, dentistry students at NGU are introduced to infection control measures via group work sessions (2 sessions along the 5-years-dentistry studying, 2 h each). Students learn infection control measures by doing in clinical dentistry classes, where they practice dentistry under supervision of faculty staff and instructors. After COVID-19 pandemic, the university held seminars to increase awareness on COVID-19 modes of infection and respiratory etiquette for all students. Per the teaching curricula, there was an addition of a learning material on oral manifestation of COVID-19 patients, yet no curricula were added regarding the infection control measures for dentists specifically, or regarding safer dental practice in the pandemic.

### Sampling methods and sample size

Sample size calculation was performed via Epi Info, and it was assumed that 50% of the dental students adopted satisfactory infection control measures during their clinical dental classes [13]; with confidence level 95% and margin of error 10%, a sample size of 97 participants was found to be enough to achieve study objectives. A total of 104 students were recruited by a non-random convenience sample.

### Study tool

An anonymous online self-administered questionnaire was used. The questionnaire was constructed in English language. The questionnaire was constructed based on "safe dental practices during COVID-19" and after reviewing previous literature [14–16]. The questionnaire included the following sections: sociodemographic aspects**,** knowledge about safe dental practice during the COVID-19 pandemic**,** adapted infection control measures in dental practice during the COVID-19 pandemic, and the dentists’ concerns regarding COVID-19 transmission during practice. A practice score was calculated as the sum of 8 questions concerning safe dental practice. The items were in 3-dimensions (all the time = 2, sometimes = 1, and never = 0); a higher score indicates better safe dental practice. Students who scored 8 or more were considered to have safe dental practices during the COVID-19 pandemic. An attitude score was calculated as the sum of 11 questions assessing students’ beliefs about the effectiveness of safe dental practices (items shown. The questions are three dimensions, ranging from 0 to 2, where higher scores reflect a more positive attitude toward safe dental measures. Students who scored 11 or more points were considered to have positive attitude toward safe dental practices. A pilot study to test questionnaire accuracy and comprehensibility was conducted on 15 dental students. Minor changes were adopted on the questionnaire after the pilot study, as rephrasing the questions and enlisting more answer options. The pilot study participants were not included in the study results.

### Statistical analysis

The collected data was analysed using the Statistical Package for Social Science (SPSS version 24) program. A suitable analysis was done according to the type of data obtained for each parameter. The Qualitative data was presented in the form of numbers and percentages (proportions). The quantitative data was presented in the form of means and standard deviations.

### Ethics approval

Participation in the study was entirely voluntary. The online questionnaire was anonymous, ensuring that no personal or identifiable information was collected. Informed consent was obtained from all participants through a consent statement provided at the beginning of the questionnaire, as well as a disclaimer that the collected data are for research purposes and will be published, and explanation of the research. Confidentiality of all collected data was strictly maintained throughout the study. Approval from the Ethical Committee at NGU was obtained (N.10.2023).

## Results

The study included a total of 104 dental students who attended clinical sessions. The participated dentistry students were in the mainly from year years 4 and 3 at the time of the study (52 (50%) and 32 (30.8%), respectively) (Table 1). The most common source of information was the school of dentistry itself (88.5%), followed by World Health Organization fact sheets (68.3%) (Table 2).

**Table 1:** Sociodemographic characteristics of the studied population (*n*= 104)

	Mean	SD	Min.	Max.
Age	21.11	1.014	19	23
			Number	%
Sex	Male		42	40.4%
	Female		62	59.6%
Academic Year	3rd year		32	30.8%
	4th year		52	50.0%
	5th year		20	19.2%

**Table 2:** Knowledge regarding safe dental practices during COVID-19 pandemic

Source of information for “safe dental practices” during the COVID-19^a^	Yes (*n*%)
School of dentistry	92 (88.5%)
World Health Organization fact sheets	71 (68.3%)
The Egyptian Ministry of Health	69 (66.3%)
Published research	48 (46.2%)
The Egyptian Dentistry Syndicate	37 (35.6%)
Center for Disease Control and Prevention	35 (33.7%)

^a^Items are not mutually exclusive

Per the adopted safe dental measures among the students on their clinical classes, the study shows that additional hand hygiene was the most practiced dental infection control precautionary measures to ensure safe dental practices during the COVID-19 pandemic, with 82.7% of the respondents reporting practicing it all the time. The least adopted measure was checking patient temperature, with only 28.8% of the respondents doing so all the time (Table 3).

**Table 3:** Practice of safe dental measures during COVID-19

Items of Safe dental practice score	All the time	Sometimes	Never
	*N* (%)	*N* (%)	*N* (%)
Additional hand hygiene	86 (82.7%)	17 (16.3%)	1 (1.00%)
Patient masks in waiting area	82 (78.8%)	16 (15.4%)	6 (5.8%)
Wearing Face shield	49 (47.1%)	50 (48.1%)	5 (4.8%)
Avoidance of aerosol-generating treatments	43 (41.3%)	40 (38.5%)	21 (20.2%)
Wearing double gloves	42 (40.4%)	44 (42.3%)	18 (17.3%)
Room rotation^*^	41 (39.4%)	29 (27.9%)	34 (32.7%)
Wearing higher masks as KN95, N95, FFP2	37 (35.6%)	51 (49.0%)	16 (15.4%)
Checking patient temperature	30 (28.8%)	34 (32.7%)	40 (38.5%)

^*^Rotating practice between two rooms to limit exposure

**Fig. 1:**
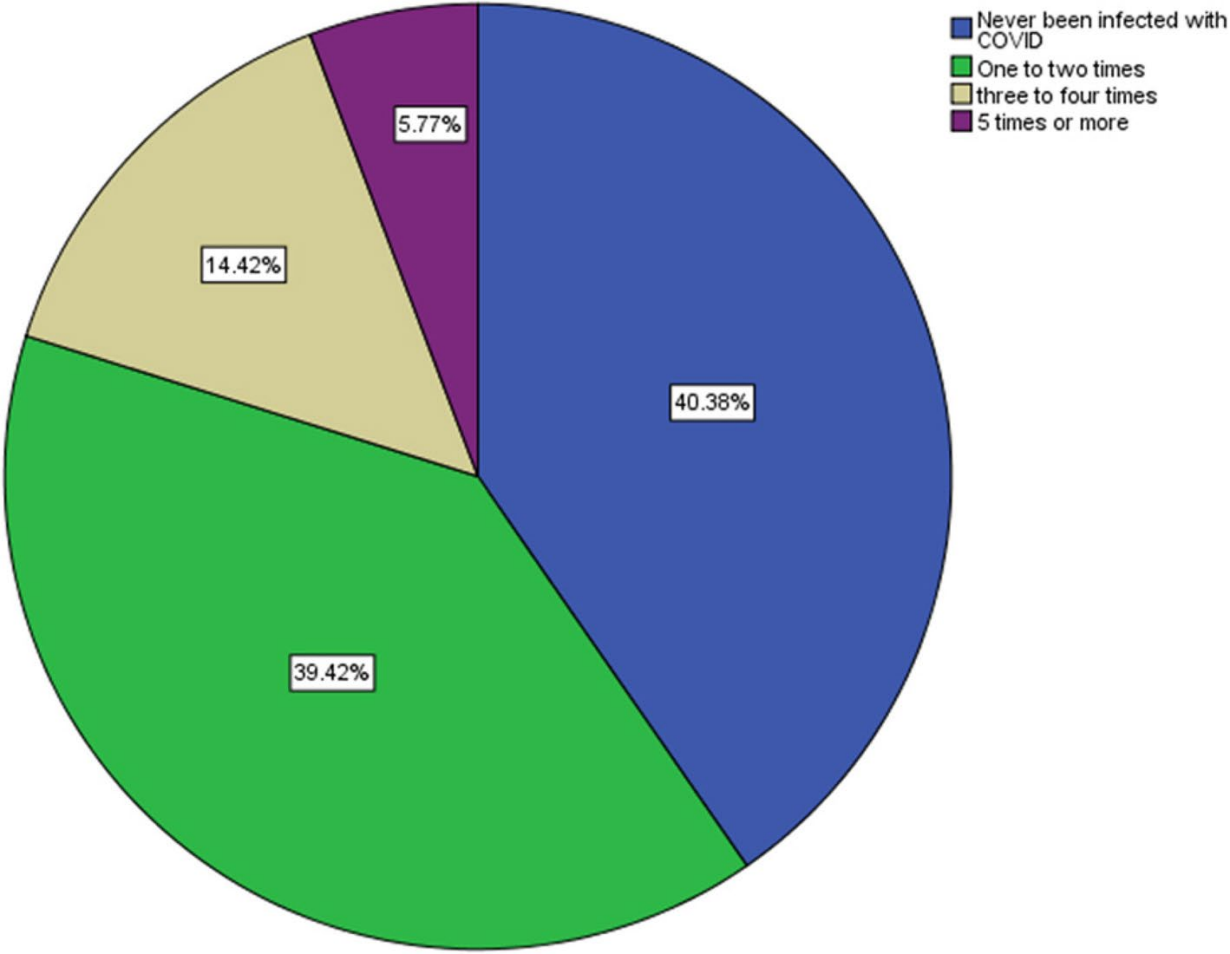
Frequency of COVID-19 infection during the past 12 months

**Fig. 2:**
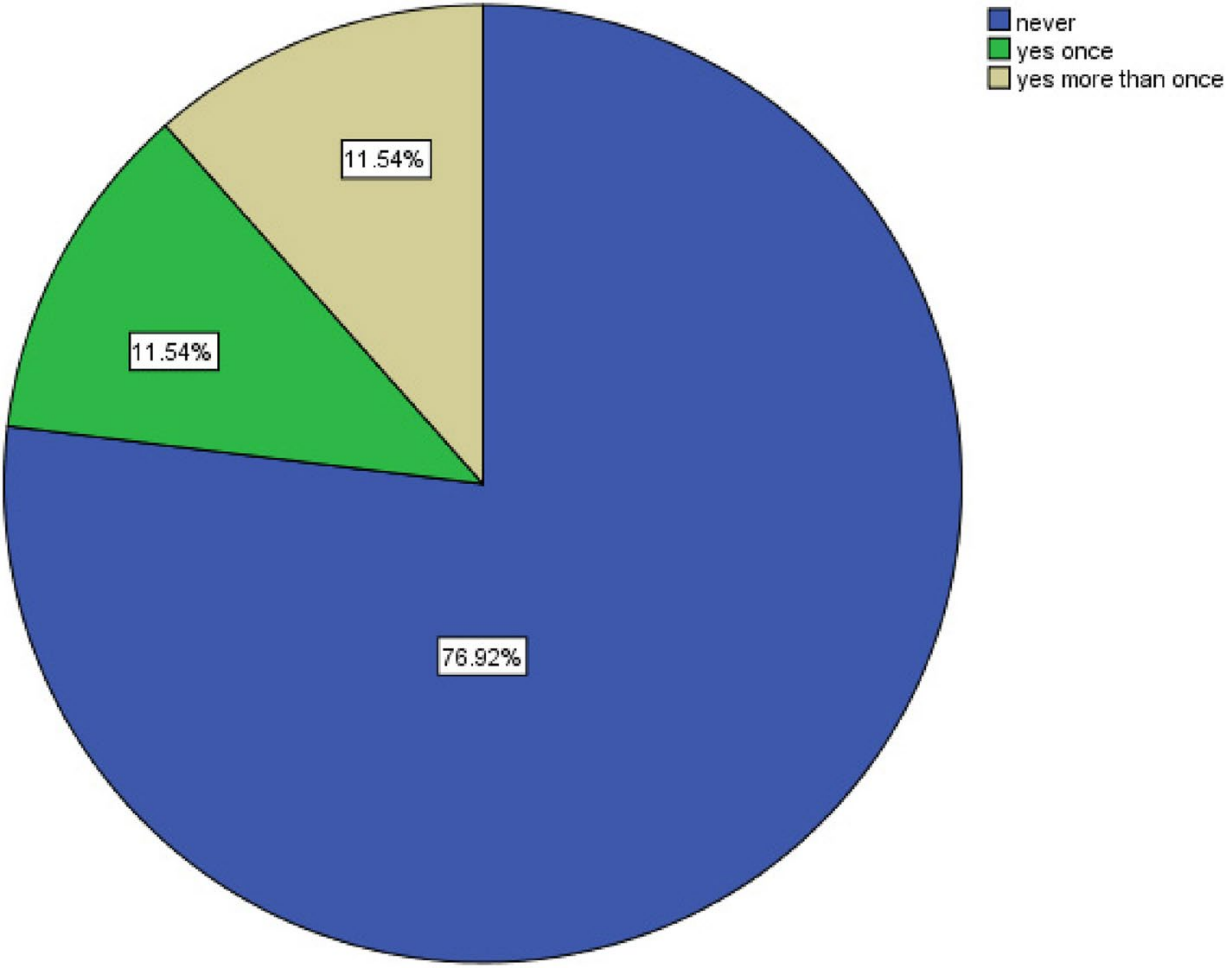
Frequency of skipping dental classes for fear of COVID-19 infection

Most students believed that the most effective measures for infection control during dental practice is wearing face shields, the patient wearing masks in the waiting area, and additional hand hygiene (Tables 4). Nearly two-thirds of the students believed that their knowledge of safe dental measured were good (Table 5). A total of 88.5% of the dental students had a satisfactory safe dental practice, and 93.2% of the students had a positive attitude towards safe dental measures. There was no statistically significant difference between male and female attitudes toward safe dental measures nor safe dental practice score (Table 6). There was a statistically significant association between age, and satisfactory safe dental practice score (Tables 7). There was a non-statistically significant in the source of knowledge of COVID-19 safe dental measures among male and female students (Table 8). There was a statistically significant association between both practicing hand hygiene and checking patient temperature, and gender (*p* = 0.023, *p* = 0.046) respectively (Table 9). There was no statistically significant association between students’ attitude toward safe dental and gender (Table 10). Female students perceived dental practice during COVID-19 as a high risk compared to male students, the difference was statistically significant (*p* = 0.003). Female students were more concerned compared to male students to get infected with COOVID-19 due to their dental practice (× 2 = 3.914, *p* = 0.048) (Table 11). Nearly 40% of the students stated that they have never been infected with COVID-19 before (Fig. 1). Nearly 77% of the students have never missing clinical classes due to fear of COVID-19 infection (Fig. 2).

**Table 4:** Students’ attitude towards safe dental measures during COVID-19

Items of Safe dental attitude score	Very effective	Neutral	Not Effective
	*N* (%)	*N* (%)	*N* (%)
Patient masks in waiting area	75 (72.1%)	27 (26.0%)	2 (1.9%)
Wearing Face shield	70 (67.3%)	30 (28.8%)	4 (3.8%)
Additional hand hygiene	69 (66.3%)	33 (31.7%)	2 (1.9%)
Avoidance of aerosol-generating treatments	62 (59.6%)	36 (34.6%)	6 (5.8%)
Checking patient temperature	57 (54.8%)	38 (36.5%)	9 (8.7%)
Wearing higher masks as KN95, N95, FFP2	56 (53.8%)	42 (40.4%)	6 (5.8%)
Additional sterilization using UV light lamps	53 (51.0%)	39 (37.5%)	12 (11.5%)
Room rotation during practice*	51 (49.0%)	40 (38.5%)	13 (12.5%)
Wearing double gloves	49 (47.1%)	38 (36.5%)	17 (16.3%)
HEPA filters	45 (43.3%)	42 (40.4%)	17 (16.3%)
Atomizer in practicing clinic	43 (41.3%)	44 (42.3%)	17 (16.3%)

^*^Rotating practice between two rooms to limit exposure

**Table 5:** Students’ beliefs and perceptions regarding dental measures and patient care during COVID-19 pandemic

		*N*	%
How do you assess risk of COVID19 infection associated with dental practice	Low	8	7.7%
	Average	30	28.8%
	High	66	63.5%
Regular dental care can be maintained during COVID-19 with the proper infection control measure	Agree	84	80.8%
	Neutral	0	0.0%
	Disagree	20	19.2%
Dental care should be only for emergency critical situations	Agree	46	44.2%
	Neutral	19	18.3%
	Disagree	39	37.5%
How do you assess your fear of Being Infected with COVID-19 due to dental practice	Concerned	46	44.2%
	Neutral	34	32.7%
	Not Concerned	24	23.1%
How do you assess your fear of infecting others with COVID-19 due to dental practice	Concerned	51	49.0%
	Neutral	38	36.5%
	Not Concerned	15	14.4%
Self-evaluation of knowledge of “safe dental Measures”	Good	70	67.3%
	Average	30	28.8%
	Poor	4	3.8%
Self-evaluation of practiced measures against COVID-19	Good	64	61.5%
	Average	36	34.6%
	Poor	4	3.8%

**Table 6:** Practice and attitude scores towards safe dental measures among male and female students

		Gender				*P* value
		Male (*n*= 42)		Female (*n*= 62)		
		N	%	*N*	%	
Safe Dental Measures Practice Score	Satisfactory (*n*= 92)	37	88.10%	55	88.70%	0.579
	Unsatisfactory (*n*= 12)	5	11.90%	7	11.30%	
Safe Dental Measures Attitude Score	Positive (*n* = 97)	39	92.90%	58	93.50%	0.593
	Negative (*n*= 7)	3	7.10%	4	6.50%

**Table 7:** Association between students’ practice and attitude towards safe dental measures and age

		Age In Years		*P* value
		Mean	SD	
Safe Dental Measures Practice score	Satisfactory (*n*= 92)	20.99	0.883	0.013
	Unsatisfactory (*n* = 12)	21.75	0.866	
Safe Dental Measures Attitude Score	Positive (*n*= 97)	21.07	0.904	0.870
	Negative (*n* = 7)	21.14	1.069

**Table 8:** Sources of information for safe dental measures during COVID-19 among male and female students

		Gender				*P* value
		Male (*n*= 42)		Female (*n*= 62)		
		*N*	%	*N*	%	
NGU school of dentistry	Yes	36	85.70%	56	90.30%	0.539
The Egyptian ministry of health	Yes	26	61.90%	43	69.40%	0.43
The Egyptian dentistry syndicate	Yes	16	38.10%	21	33.90%	0.659
WHO fact sheets	Yes	26	61.90%	45	72.60%	0.251
CDC	Yes	14	33.30%	21	33.90%	0.955
Published research	Yes	20	47.60%	28	45.20%	0.805

**Table 9:** Practicing safe dental measures during COVID-19 among male and female students

		Gender				*P* value
		Male (*n*= 42)		Female (*n*= 62)		
		*N*	Column %	*N*	Column %	
Wearing Face shield	All the time	18	42.90%	31	50.00%	0.087
	Sometimes	21	50.00%	29	46.80%	
	Never	3	7.10%	2	3.20%	
Checking patient temperature	All the time	12	28.60%	18	29.00%	0.046
	Sometimes	19	45.20%	15	24.20%	
	Never	11	26.20%	29	46.80%	
Wearing double gloves	All the time	15	35.70%	27	43.50%	0.083
	Sometimes	19	45.20%	25	40.30%	
	Never	8	19.00%	10	16.10%	
Wearing higher masks as KN95, N95, FFP2	All the time	14	33.30%	23	37.10%	0.089
	Sometimes	20	47.60%	31	50.00%	
	Never	8	19.00%	8	12.90%	
Additional hand hygiene	All the time	30	71.40%	56	90.30%	0.023
	Sometimes	12	28.60%	5	8.10%	
	Never	0	0.00%	1	1.60%	
Patient masks in waiting area	All the time	33	78.60%	49	79.00%	0.135
	Sometimes	6	14.30%	10	16.10%	
	Never	3	7.10%	3	4.80%	
Avoidance of aerosol-generating treatments	All the time	16	38.10%	27	43.50%	0.075
	Sometimes	22	52.40%	18	29.00%	
	Never	4	9.50%	17	27.40%	
Room Rotation	All the time	16	38.10%	25	40.30%	0.09
	Sometimes	14	33.30%	15	24.20%	
	Never	12	28.60%	22	35.50%

**Table 10:** Attitude towards effectiveness of the following measures for a safe dental practice during COVID among male and female students

		Gender				
		Male (*n*= 42)		Female (*n*= 62)		
		*N*	%	*N*	%	*P* value
Wearing Face shield	Very Effective	26	61.9%	44	71.0%	0.342
	Neutral	14	33.3%	16	25.8%	
	Not Effective	2	4.8%	2	3.2%	
Wearing double gloves	Very Effective	18	42.9%	31	50.0%	0.288
	Neutral	15	35.7%	23	37.1%	
	Not Effective	9	21.4%	8	12.9%	
Wearing higher masks as KN95, N95, FFP2	Very Effective	23	54.8%	33	53.2%	0.950
	Neutral	16	38.1%	26	41.9%	
	Not Effective	3	7.1%	3	4.8%	
Additional hand hygiene	Very Effective	26	61.9%	43	69.4%	0.240
	Neutral	14	33.3%	19	30.6%	
	Not Effective	2	4.8%	0	0.0%	
Additional sterilization using UV light lamps	Very Effective	18	42.9%	35	56.5%	0.186
	Neutral	18	42.9%	21	33.9%	
	Not Effective	6	14.3%	6	9.7%	
Atomizer in practicing clinic	Very Effective	17	40.5%	26	41.9%	0.332
	Neutral	15	35.7%	29	46.8%	
	Not Effective	10	23.8%	7	11.3%	
HEPA filters	Very Effective	17	40.5%	28	45.2%	0.236
	Neutral	15	35.7%	27	43.5%	
	Not Effective	10	23.8%	7	11.3%	
Checking patient temperature	Very Effective	23	54.8%	34	54.8%	0.423
	Neutral	18	42.9%	20	32.3%	
	Not Effective	1	2.4%	8	12.9%	
Patient masks in waiting area	Very Effective	31	73.8%	44	71.0%	0.544
	Neutral	11	26.2%	16	25.8%	
	Not Effective	0	0.0%	2	3.2%	
Avoidance of aerosol-generating treatments	Very Effective	24	57.1%	38	61.3%	0.839
	Neutral	16	38.1%	20	32.3%	
	Not Effective	2	4.8%	4	6.5%	
Room rotation during practice	Very Effective	24	57.1%	27	43.5%	0.182
	Neutral	14	33.3%	26	41.9%	
	Not Effective	4	9.5%	9	14.5%

**Table 11:** Beliefs and perceptions regarding dental measures and patient care during COVID-19 pandemic among male and female students

		Gender				*P* value
		Male (*n*= 42)		Female (*n*= 62)		
		*N*	%	*N*	%	
Risk of COVID19 infection associated with dental practice	Low	7	16.7%	1	1.6%	
	Average	14	33.3%	16	25.8%	0.003
	High	21	50.0%	45	72.6%	
Regular dental care can be maintained during COVID-19 with the proper infection control measure	Agree	15	35.7%	31	50.0%	0.641
	Neutral	13	31.0%	21	33.9%	
	Disagree	14	33.3%	10	16.1%	
Dental care should be only for emergency critical situations	Agree	18	42.9%	33	53.2%	
	Neutral	15	35.7%	23	37.1%	0.484
	Disagree	9	21.4%	6	9.7%	
How do you assess your fear of Being Infected with COVID-19 due to dental practice	Concerned	27	64.3%	43	69.4%	
	Neutral	15	35.7%	15	24.2%	0.048
	Not Concerned	0	0.0%	4	6.5%	
How do you assess your fear of infecting others with COVID-19 due to dental practice	Concerned	29	69.0%	35	56.5%	0.125
	Neutral	12	28.6%	24	38.7%	
	Not Concerned	1	2.4%	3	4.8%	
Self-evaluation of knowledge of “safe dental Measures”	Good	19	45.2%	27	43.5%	
	Average	10	23.8%	9	14.5%	0.901
	Poor	13	31.0%	26	41.9%	
Self-evaluation of the quality of practiced measures against COVID-19	Good	33	78.6%	51	82.3%	
	Average	0	0.0%	0	0.0%	0.186
	Poor	9	21.4%	11	17.7%

## Discussion

This research provides insight not only into safe dental practices during the COVID-19 pandemic but also, into the standard infection control measures needed for safe dental practice. Dentists are at risk of becoming infected or infecting patients during their practice [6]. On a daily basis, dentists are in contact with multiple sources of infection, such as the patient's saliva, contaminated materials, and dental surfaces [17]. Dental practice during the pandemic subsequently increased the risk of COVID-19 transmission. These findings demonstrate the importance of possessing sufficient knowledge and training not only to protect against COVID-19 but also as a guideline for safe dental practice in general. The current study revealed that more than half of the dental students self-evaluated their safe dental measures during clinical dental classes as “good” (61.5%).

With respect to the students’ sources of information, the main sources of information on safe dental practices during the COVID-19 pandemic were the school of dentistry itself and the World Health Organization website, which are credible medical sources and trusted governmental institutions, confirming that the students had reliable high-quality information regarding safe dental practices. A study conducted in six sub-Saharan countries reported that more than half of participants relied on social media as a source of COVID-19-related information and that those who relied on social media as a source of information agreed more readily with the effectiveness of masks than did those who did not rely on social media (AOR 1.33, 95% CI: 1.01–1.77) [18]. Moreover, a study conducted among a group of Ethiopian healthcare workers reported that nearly half of the participants relied on social media as a source for COVID-19 information [19], which proposed social media as a good source of safe dental practice information. Yet, a study conducted in Jordan reported that most medical students (83.4%) relied on social media to gain information about COVID-19; however, only 9.7% of the students used face masks routinely, and only 19.3% believed that face masks were effective [20]. These findings suggest that the source of information has an impact on the safety of dental knowledge, attitude, and practice of students. Social media, as a source of health information, has both advantages and advantages. It can be a communication tool and a portal for international and governmental health organizations to offer valid and reliable health information. However, social media lacks an accuracy checker to validate the information. Moreover, social media algorithms suggest content based on user’s activity. This may lead to the dissemination of false knowledge and rumors. While social media offers a tool to disseminate information, its use in health should be used with caution and not as a replacement for medical school and professional organization information. In the current study the students primarily relied on the knowledge disseminated through the school itself regarding COVID-19. As part of their regular dentistry curricula, dentistry students at NGU are introduced to infection control measures via group work sessions (2 sessions along the 5-years-dentistry studying, 2 h each). Students were taught the infection control measures in clinical dentistry classes, where they practiced dentistry under supervision of faculty staff and instructors. After COVID-19 pandemic, the university held seminars to increase awareness on COVID-19 modes of infection and respiratory etiquette for all students, which seemed satisfactory for the students as (67.3%) and (61.5%) self-evaluated their knowledge and practice of safe dental measures as good. Moreover, the current study showed that most of the dental students had a positive attitude score as well as a satisfactory practice score of safe dental measures. Moreover, nearly 40% of the students stated that they have never been infected with COVID-19. However, some area needed improvement, and more effort is needed to raise students’ awareness of the importance of measuring patient temperature, using advanced sterilization techniques, especially that the students reported that they were only and added teaching session during the pandemic on oral manifestation of COVID-19 patients, yet no curricula were added regarding the additional infection control measures dentists specifically should apply to have a safe practice during the pandemic.

Since the emergence of the COVID-19 pandemic, attention has been given to dental practices, and precautionary measures have been implemented. This study highlighted concerns related to the increased risk of infection associated with dental practices. Many studies have shown a growing tendency to limit dental practice to emergency cases only during the pandemic. In a study conducted in Brazil, 64.6% of dentists attended only emergency treatments during the pandemic [21]. Similarly, a study in China revealed that patients themselves were reducing their visits to the dentist, with a 70% reduction in patients seeking nonurgent dental services during the pandemic [22]. Moreover, a study conducted in Egypt among dental students at a governmental university reported that 68.2% of the students wished that they could stop their dental practice during the pandemic [13]. These results reflect the shared concern that dental procedures pose a high risk of transmission worldwide. The participants in the current study also reported concerns about becoming infected or infecting others during their practice. This finding is consistent with another study in which dentists feared contracting COVID-19 due to their profession and feared infecting their family members [23]. Results of the present study showed that gender influenced the students’ tendency to abide by infection control and safety measures. With respect to safe dental practices during the COVID-19 pandemic, most female students practiced hand hygiene (90.3%), whereas (71.4%) of male students did. Similarly, a study conducted in Saudi Arabia, showed that female dental students had higher compliance with safe dental practices [24]. The results of the present study also revealed that only (26.2%) of the male students “never” checked patient temperature, whereas (46.8%) of the female students did. These results contradict those of Younis et al. [13] who reported a statistically nonsignificant association between sex and safe dental practice scores.

The results of the present study revealed that age was associated with having a satisfactory safe dental measures practice score. Similarly, previous study revealed that more senior students than junior students had safe dental practice scores [13]. A study in Turkey reported that clinical students had higher health anxiety inventory scores than non-clinical students did; however, the study did not report any difference in anxiety scores across different age groups [25]. This may be associated with senior students feeling less concerned, more experienced, or more confident, which could be linked to a more relaxed approach and less strict adherence to infection control and safe dental practice measures. However, the present study did not find any statistically significant difference in the level of students’ concern and fear of COVID-19 according to academic year or age.

On the NGU campus, COVID-19 vaccination campaigns were launched during the 2021–2022 academic year, as COVID-19 vaccination was mandatory for school enrollment, and all students were vaccinated with the COVID-19 vaccine. The results of the present study revealed that most of the students reported being infected with COVID-19, as 41% of the respondents experienced one to two COVID-19 infections. These rates are expected since the field of dentistry requires close contact with patients, thereby increasing the risk of infection transmission. The COVID-19 infection rates reported in this study are similar to rates reported among medical students in a study conducted in Jordan, where 34.6% of the students reported COVID-19 infection [26]. This highlights that routine infection control measures as hand wash are just not enough alone for prevention of COVID-19 infections, as 38.5% of the students never checked for patient temperature, and 32.7% never adopted room rotation to avoid risk of COVID-19 as advised in the guidelines [27].

More than half of the students (68.3%) believed that their safe dental practice knowledge was “good or very good”. This could be explained by the infection control protocols mandated by the university itself. The School of Dentistry protocol prohibits students from working in labs without proper use of personal protective equipment PPE. The results also revealed that fewer than one-third of the students had skipped clinical classes due to fear of infection; moreover, 76.9% of the students never skipped practical sessions in fear of becoming infected. This could be due to their trust in PPE; yet this may also be due to the university’s strict attendance policies.

The current study revealed that the most common practiced safety measure used hand hygiene (82.7%). This reflects individuals’ dedication to limiting the spread of COVID-19, especially as hand hygiene is the most feasible and efficient method to prevent transmission [28]. The current study revealed that the students were aware of the high risk of infection associated with dental practice, as 63.5% of the students believed that the risk of infection during dental practice was high, which reflects a high awareness of hazards associated with dental practice among the students. Unexpectedly, checking patients’ temperature was the least safe dental practice measure adopted among the students, and only 28.8% of the students reported practicing it. Checking a patient’s temperature is a safety measure that can be performed quickly and regularly, which is why it is expected to be done as frequently as the hand hygiene. Not measuring the temperature of the patients, nor taking history of fever or use of antipyretics can strongly increase the risk of infection [29].

The current study revealed that 16.3% of the students believed that HEPA filters and using atomizer were not effective measures for safe dental practice. This is an unexpected result since the use of HEPA filters is an exceptional method for preventing air borne infections. The filters remove bioaerosols from the air, eliminating contaminants [30]. Moreover, 16.3% of the current study participants believed that wearing double gloves was not effective in reducing infections among dentists. Similarly, a study conducted in India among interns, dental postgraduate students, and dental faculty members reported that 5.2%, 12.1%, and 12.7% of the participants, respectively, believed that wearing double gloves was not effective in reducing infections [31]. Dentists are exposed to pathogens during their practice and wearing double gloves reduces the risk of microorganism transmission for safer practice [32, 33]. This highlights that students’ attitude toward necessity and effectiveness of wearing gloves during dental practice still need further improvement. The current study showed that female dental students were more concerned about the risk of COVID-19 infection and subsequently more adherent to most of the precautionary infection control measures compared to their male colleagues. This finding is consistent with a previous study conducted in Egypt which reported that female dentists were more adherent to COVID-19 infection control measures [34].

More than one third (35.6%) of the students used (KN95, N95, FFP2) masks “all the time”, and 50% used them “sometimes” during their practice. This finding aligns with that of Ameena et al. [35], who surveyed dental practitioners, dental house surgeons, postgraduate students, and teaching faculty, and reported that nearly half of them (54.7%) used N95 masks while performing aerosol-generating procedures and that 10.3% of the study participants used both regular masks and N95 masks [35]. Only 53.8% of the studied students believed that wearing the before mentioned types of masks was an effective precautionary measure for safe dental practice. This is true, as using these masks was the most efficient solution to prevent infection as revealed in a study that analyzed coughing and COVID-19 transmission [36]. Similarly, a study conducted among specialized dentists revealed that more than three fourths of the specialists (78.7%) believed that the N-95 mask was effective in protecting against COVID-19, and 67.3% believed hand washing to be effective [37]. Additionally, a study of Jordanian dentists showed that, most of them (92.2%) were compliant with safe dental practice measures as goggles, masks, and gloves [11]. Compared with the students in our study, the increase in age and dental years of experience improved dentists’ knowledge and perceptions of the adopted precautionary measures.

## Conclusions and Recommendations

The current study highlights key strengths and gaps in dental education, reinforcing the importance of safety protocols in enhancing professional standards and safeguarding public health. The current study showed that most dental students were aware of preventive measures, demonstrated a positive attitude and reported incorporating them into clinical dental sessions during the COVID-19 pandemic. The infection control practices adopted during this period may serve as a foundation for enhancing routine dental infection control protocols. Nevertheless, there is still room for improvement, a more structured and detailed curriculum on infection control measures may be beneficial—particularly in areas such as consistent patient temperature monitoring and improving students’ understanding and attitude toward the use of HEPA filters. Incorporating a mandatory patient temperature screening is warrented. Future research, especially multicenter studies involving governmental dental schools and practicing dentists, could provide broader insights and help shape interventions to strengthen safe dental practices.

## Limitations

This study has several limitations. It was conducted at a single private dental school with a relatively small sample size, which may limit generalizability—particularly to public dental faculties with different resources and conditions. The use of convenience sampling may have introduced selection bias, as participants who were more available or willing to respond may not represent the broader student population. Data were collected through self-reported questionnaires, which are subject to recall and social desirability biases, and students’ actual clinical behaviour was not confirmed by observation. These may be associated with overreporting of positive behaviors or attitude.

## Data Availability

No datasets were generated or analysed during the current study.

## Abbreviations

COVID-19: Coronavirus Disease of 2019
FFP2: Filtering Face Piece
HEPA: High efficiency particulate air
KN95: A KN95 is a mask that is built to Chinese standards issued by the Standardization Administration of the People's Republic of China
N95: A particulate-filtering respirator that meets the US National Institute for Occupational Safety and Health (NIOSH)
PPE: Personal protective equipment
